# Homeobox protein VentX induces p53-independent apoptosis in cancer cells

**DOI:** 10.18632/oncotarget.9238

**Published:** 2016-05-09

**Authors:** Hong Gao, Bin Wu, Yi Le, Zhenglun Zhu

**Affiliations:** ^1^ Department of Medicine, Brigham and Women's Hospital, Harvard Medical School, Boston, 02115, Massachusetts, USA; ^2^ Current address: Department of Medicine, Tufts Medical Center, Boston, 02115, Massachusetts, USA; ^3^ Current address: Department of Gastroenterology, Third Hospital, Sun Yat-Sen University, Guangzhou, 510630, China

**Keywords:** ventX, apoptosis, p53, caspase-3, PARP

## Abstract

Identifying novel tumor suppressors holds promise for improving cancer treatment. Our recent studies identified VentX, a homeobox transcriptional factor, as a putative tumor suppressor. Here we demonstrate that VentX exerts strong inhibitory effects on the proliferation and survival of cancer cells, but not primary transformed cells, such as 293T cells. Mechanistically, both *in vitro* and *in vivo* data showed that VentX induces apoptosis of cancer cells in a p53-independent manner. We found that VentX expression can be induced by chemotherapeutic agents. Taken together, our findings suggest that VentX may function as a novel therapeutic target in cancer treatment.

## INTRODUCTION

Uncontrolled proliferation and inadequate differentiation are hallmarks of cancers. Thus anti-proliferation and pro-differentiation factors serve as ideal targets to slow cancer progression. However, the nature of such factors and their potential applications in cancer therapy remain to be further defined.

Developmental modeling serves as a unique tool to identify factors involved in cell proliferation and differentiation. Dorsoventral axis formation is the earliest step in cell differentiation during early embryogenesis [1]. Beta-catenin of the canonical Wnt pathway is a key component of the dorsal signaling center, which drives the development of dorsal cell fate [2–4]. Beta-catenin exerts its effects through activating the HMG-box containing LEF/TCF factors [5] [6, 7]. The effect of beta-catenin in dorsalization is counteracted by BMP4 signaling of the ventral signaling center [8]. The homeobox protein Xom/Vent2 is the major mediator of BMP4 signaling during early vertebrate embryogenesis [9, 10]. In trying to understand how dorsoventral signaling is integrated to dictate cell fate, we found Xom/Vent2 of the BMP4 signaling pathway to be a LEF/TCF-associated Wnt antagonist [11]. Given the prominent role of canonical Wnt signaling in oncogenesis, we tried to translate this basic finding into potential clinical relevance by searching for the human homologue of Xom/Vent2. This effort identified VentX, a human Xom/Vent2 homologue, as a novel antagonist of canonical Wnt signaling [12, 13]. We found that VentX controls the proliferation and differentiation of human hematopoietic cells of both myeloid and lymphoid lineages. We showed that VentX exerts it functions, in part, by activating p53/p21 and p16/pRB pathways [14] [15]. Expression analysis showed that VentX expression is reduced in lymphocytic leukemia [13], which led to the hypothesis that VentX functions as a tumor suppressor in lymphocytic leukemia [13, 15]. VentX resides on chromosome 10q26.3, distal to the PTEN tumor suppressor gene, a “hot region” that is implicated in all cancer types [16]. As both canonical Wnt signaling and the p53 pathways have been implicated in the pathogenesis of hematological malignancies as well as solid tumors [17, 18], we asked whether VentX exerts a tumor-suppressive effect on solid tumors.

The current study shows that VentX exerts strong inhibitory effects on the proliferation and survival of cancer cells derived from solid tumors. In comparison, VentX exerts little inhibitory effect on the survival of primary transformed cells, such as 293T cells. Mechanistically, both *in vitro* and *in vivo* data show that VentX induces apoptosis in cancer cells bearing either wild-type or mutated p53. VentX expression can be induced in solid tumor cells by chemotherapeutic agents, suggesting that VentX may function as a novel therapeutic target.

## RESULTS

### *VentX* encodes a nuclear protein

**Figure 1:**
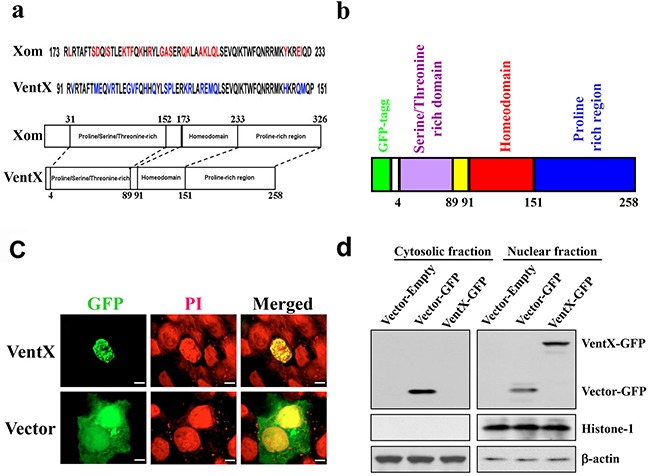
VentX encodes a nuclear protein **a.** Schematic alignment of the homeobox and functional domains of Xom and VentX. **b.** Schematics of GFP-VentX constitutive expression construction. **c.** VentX encodes a nuclear protein. Constructs encoding GFP-VentX or GFP were transfected into HCT116 cells. Twenty-four hours post-transfection, the cells were fixed, and the nuclei were counterstained with propidium iodide (PI). These cells were then analyzed by confocal microscopy. **d.** The sub-cellular distribution of GFP-VentX and GPF was determined by western blotting using an anti-GFP antibody. Histone-1 and β-actin were used as loading controls for the nuclear and cytoplasm fractions, respectively. Note: VentX was observed in the nuclear fraction, but not in the cytosolic fraction.

Gain-of-function mutations in the Wnt signaling pathway have been implicated in the pathogenesis of a variety of solid tumors, such as colorectal cancers, prostate cancers, and lung cancers. Using reverse-genetic approaches, our recent studies identified VentX as a novel Wnt antagonist and a putative tumor suppressor in hematopoietic malignancies. To determine the potential effects of VentX on solid tumors, we sought to determine its intracellular distribution and effects in HCT116 colon cancer cells. Constructs encoding GFP-VentX or GFP were transfected into HCT116 cells, and the intracellular distribution of the proteins was visualized with confocal microscopy (Figure 1b and 1c). The nuclei of the HCT116 cells were labeled with propidium iodide (PI). As shown in Figure 1c, VentX is targeted to the nuclei, where it co-localizes with PI. To further verify the nuclear localization of VentX, we fractioned the sub-cellular compartments of HCT116 cells with sucrose gradient and determined the distribution of VentX in each compartment via western blot analysis. We found that VentX is enriched in the nuclear fraction of the transfected cells, while GFP is enriched in the cytoplasmic compartment of the transfected cells (Figure 1d).

### VentX suppresses human cancer cell growth and induces apoptosis

**Figure 2:**
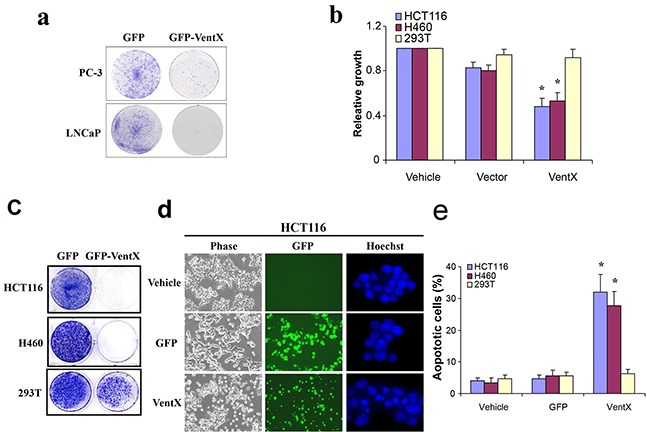
VentX suppresses the growth of human cancer cells and induces apoptosis **a.** VentX suppresses the growth of the indicated prostate cancer cells as determined by colony formation assay. **b.** VentX suppresses the growth of the indicated human cancer cells as determined by MTS assay. The growth of cells transfected with vehicle control was defined as 100%. **c.** Colony-formation assay of VentX effects on indicated cancer cells. **d.** VentX induces apoptosis. HCT116 cells were transfected with plasmids encoding GFP and GFP-VentX. Forty-eight hours post-transfection, the cells were harvested and stained with Hoechst 33528. Apoptotic cells are indicated by the characteristic features of condensed chromatin and fragmented nuclei. **e.** The percentage of apoptotic cells was calculated by counting a minimum of 400 cells, and the experiments were repeated twice with identical results. Values are mean ± SD, *P<0.01 *v.s.* vector-transfected cells.

VentX resides in chromosome 10q26, a region that is distal to the Pten, a tumor suppressor that is frequently deleted in advance prostate cancers [20]. As VentX is an antagonist of the Wnt signaling implicated in prostate cancers, we sought to determine the potential effects of VentX on the growth of prostate cancer cells. We examined the effects of VentX on proliferation and growth of PC-3 and LNCap, using a colony formation assay. As shown in Figure 2a, our data showed that ectopic expression of VentX suppressed the growth of prostate cancer cells. To determine the effects of VentX on the growth of other solid tumors in which aberrant Wnt signaling has been implicated, we tested the effects of VentX on the growth of HCT116 colon cancer cells and H460 lung cancer cells. Using colony formation assay and MTS assay, we found that VentX exerts strong inhibitory effects on the growth of these cancer cells. In comparison, VentX exerts minimal growth-inhibitory effects on transformed human embryonic kidney (HEK293T) cells (Figure 2b and 2c).

To determine the mechanisms underlying VentX-induced growth suppression, cells transfected with GFP-VentX or GFP control were examined using fluorescent microscopy. Cellular changes indicative of apoptosis, such as condensed chromatin and micronucleation [21], were noted in cells transfected with GFP-VentX. As shown in Figure 2d and 2e, the GFP did not induce cellular apoptosis, whereas GFP-VentX did induce apoptosis in 30-40% of transfected HCT116 and H460 Cells. In comparison, GFP-VentX does not induce significant apoptosis in 293T cells (Figure 2e).

### VentX induces apoptotic cell death in p53-deficient cells

**Figure 3:**
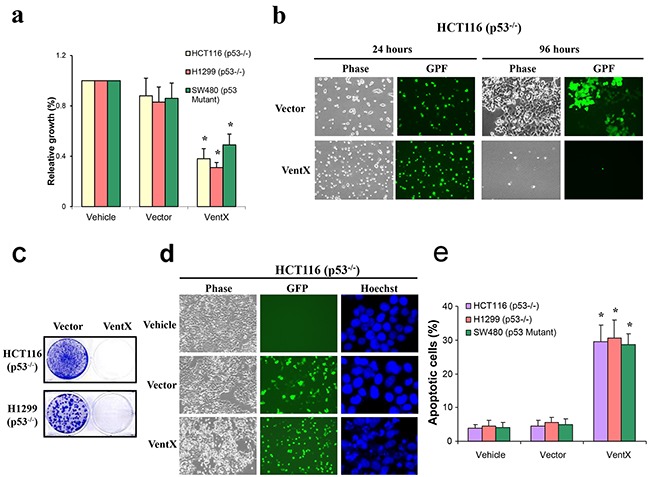
VentX induces apoptotic cancer cell death in p53 deficient cancer cells **a.** Effects of VentX on the growth of cancer cells with deficient p53 as measured by MTS assay. The growth of vehicle-treated cells was defined as 1 (100%). **b.** VentX inhibits *in vitro* growth of HCT11p53KO cells. **c.** Colony formation assay for long-term survival of indicated p53-deficient cells upon expression of VentX. **d.** VentX induces apoptosis in p53-deficient HCT116p53KO cells. **e.** The percentage of apoptotic cells upon expression of VentX was calculated by counting a minimum of 400 cells in each determination, and the experiment was repeated twice with identical results. Values are mean ± SD, *P<0.01 *v.s.* vector-transfected cells.

P53 is a critical tumor-suppressor gene. More than 50% of cancers harbor inactive p53 as a result of direct mutations in the *p53* gene [22]. For example, unbiased sequencing of whole genomes of colon cancers confirmed that *p53* is the most frequently mutated gene [23]. Likewise, *p53* mutations occur in ~50% of non-small-cell lung cancers and >70% of small-cell lung cancers [24]. Moreover, the importance of p53 is also reflected in mutations of genes downstream of p53 during carcinogenesis and cancer development. Our findings that VentX inhibits the growth of PC-3 cells, which does not contain a functional p53, prompted us to explore whether the tumor-suppression effect of VentX requires a functional p53. Thus we investigated the effect of VentX on the growth of H1299 lung cancer cells and SW480 colon cancer cells, in both of which the function of p53 has been lost due to mutations. We have also studied the effects of VentX on the growth of HCT116 p53KO, in which the functional p53 was artificially deleted. As shown in Figure 3a-3e, using MTS assay (Figure 3a), *in vitro* cell growth assay (Figure 3b), and colony formation assays (Figure 3c), we found that VentX exerts strong inhibition on the growth of these cancer cells lacking a functional p53 (P<0.01, respectively) (Figure 3d and 3e). Our data suggest that VentX induces apoptosis in cancer cells in a p53-independent manner.

### VentX induces caspase-3 activation

**Figure 4:**
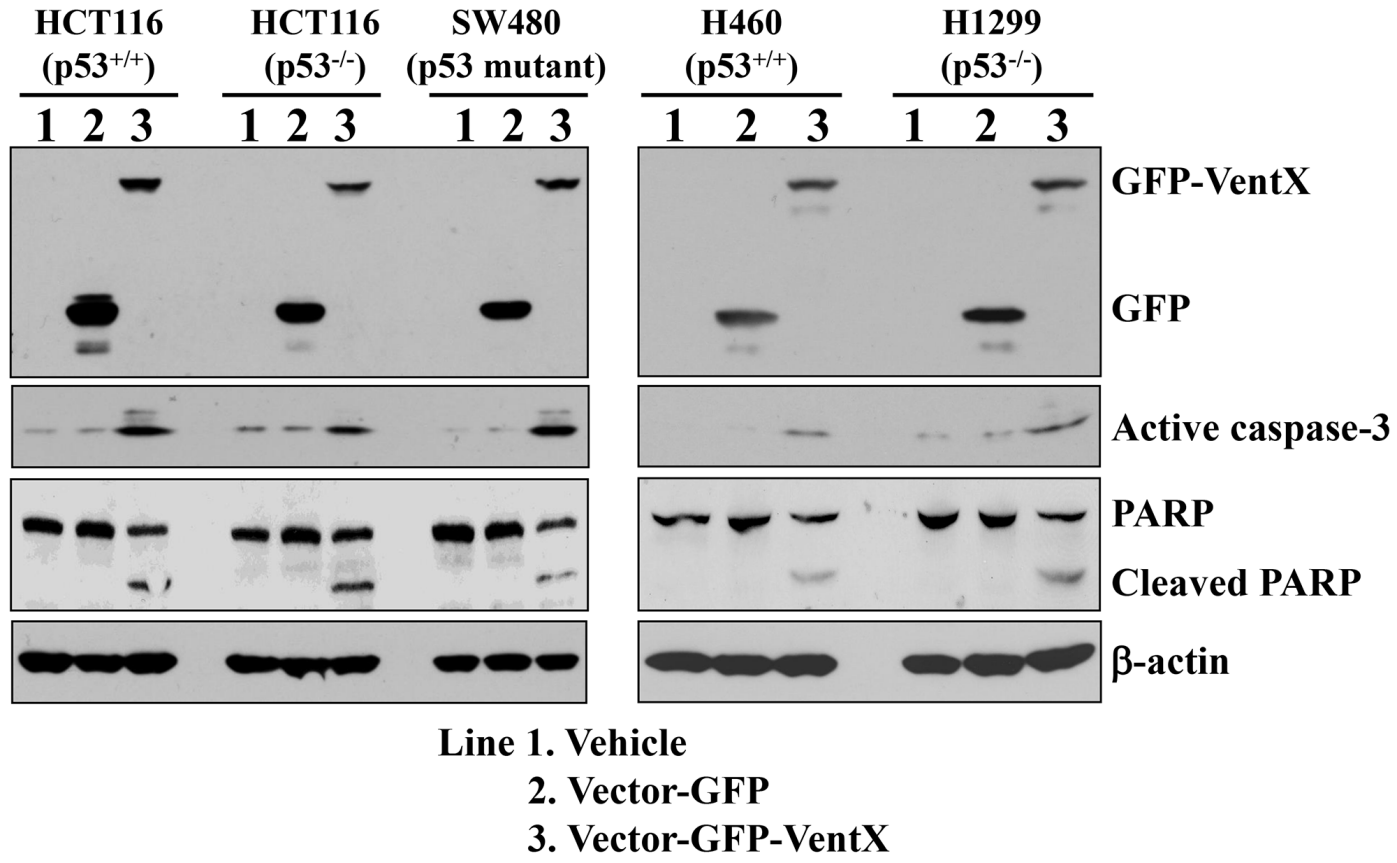
VentX induces activation of Caspase-3 in human cancer cells Indicated human cancer cells were transfected with constructs encoding GFP or GFP-VentX. Forty-eight hours post transfection, the cells were harvested and total proteins extracted. Caspase-3 activation was analyzed by western blotting assay using an active caspase-3 antibody. The cleavage of PARP was determined by specific antibody in western blotting assay. β-actin was used as the loading control.

Caspase-3 is a critical executor of apoptosis, and is either partially or totally responsible for the proteolytic cleavage of key proteins such as the nuclear enzyme poly (ADP-ribose) polymerase (PARP) [25]. Caspase-3 has been identified as a key mediator of apoptosis in mammalian cells. Induction of apoptosis leads to cleavage of procaspase-3 and the generation of an active 17-kDa caspase-3 and 12-kDa caspase-3 fragments [26]. The activated caspase-3 then targets key modulators of the apoptotic pathway including PARP and other caspases. To investigate whether caspase-3 activation is involved in VentX-induced apoptosis, HCT116 cells were transfected with plasmid encoding GFP or GFP-VentX. Forty-eight hours post-transfection, total proteins were extracted, and caspase-3 activation was analyzed by western blotting assay using an active caspase-3 antibody. As shown in Figure 4, expression of GFP-VentX but not GFP activates caspase-3 in both p53-sufficient and -deficient cancer cells. These results suggest that VentX may activate caspase-3 in a p53-independent manner.

### VentX represses tumor growth and induces apoptotic cell death *in vivo*

**Figure 5:**
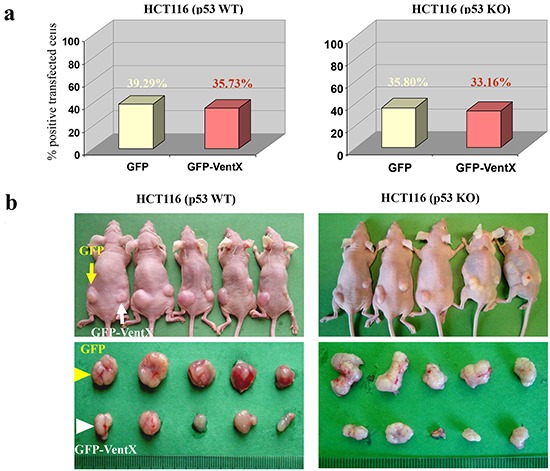

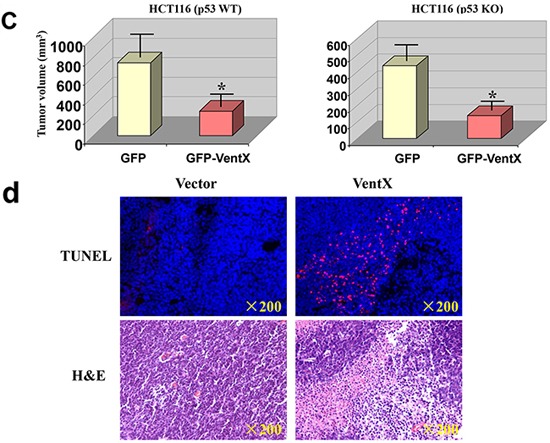
VentX represses tumors growth and induces apoptotic cell death *in vivo* **a.** Wild-type and p53 knockdown HCT116 cells were transfected with constructs encoding GFP or GFP-VentX. The transfection rate is 35-40%. **b.** Top panel shows tumor-bearing nude mice inoculated with HCT116 cells expressing either GFP (left) or GFP-VentX (right). Bottom panel shows excised tumors at the end of the experiments. **c.** Tumor growth was significantly reduced in VentX-transfected p53-WT and p53-KO HCT116 cell lines in comparison with GFP-transfected cells. Values are mean ± SD, *n*=5 per group, *P<0.01 *v.s.* vector-transfected cells. **d.** The excised xenograft tumors were fixed, embedded, and sectioned. Apoptosis was analyzed by TUNEL staining (red) with DAPI counterstaining (blue). Tumor histology was analyzed using H&E staining.

Tumor growth is affected by the microenvironment. To determine whether VentX confers antitumor activity in *vivo*, we used a xenograft model. Both HCT116 p53 wild-type (WT) cells and HCT116 p53 knockout (KO) cells were transfected with plasmids encoding GFP or GFP-VentX (the transfection rate was 35-40% (Figure 5a). The effects of VentX on tumor growth were determined by subcutaneous injection of transfected cells into nude mice. Tumor growth was examined *in situ* (Figure 5b upper panel) and as excisions (Figure 5b lower panel). We found that GFP-VentX, but not GFP, inhibits *in vivo* growth of both p53-sufficient and p53-deficient HCT116 cells (Figure 5c). H&E staining and *in situ* apoptosis analysis by TUNEL staining showed that tumors expressing VentX displayed a large amount of fragmented DNA, suggesting that VentX represses tumor growth *in vivo* by inducing apoptotic cell death (Figure 5d).

### VentX expression is induced by chemotherapeutic agents in solid tumor cells

**Figure 6:**
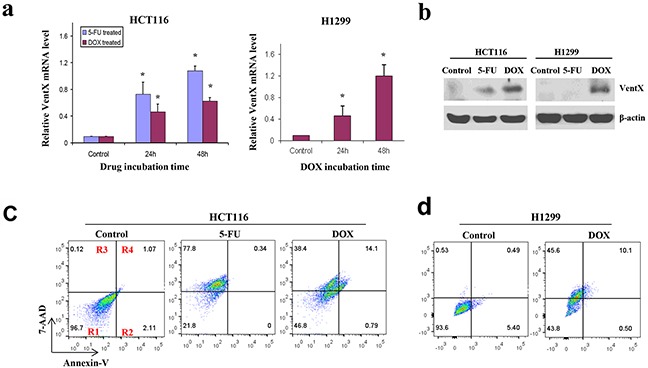
VentX expression is induced by chemoagents in solid tumor cells **a.** Quantitative RT-PCR of VentX expression in HCT116 cells and H1299 cells. Cells were incubated with 40 μg/mL of 5-FU or 1.0 μg/mL of DOX or DMSO control for indicated times. The mRNA level of VentX was determined by real-time PCR. Means ± S.D. of three independent experiments are shown, **; *P* <0.01. **b.** VentX protein levels in HCT116 and H1299 cells after 5-FU or DOX treatment as determined by western blot analysis. β-actin was used as control. **c.** Percentage of apoptotic HCT116 after 5-FU or DOX treatment as determined by annexin V and 7-AAD staining. **d.** Percentage of apoptotic H1299 cells after DOX treatment. The percentage of viable cells (region R1), early apoptotic cells (region R2), and late apoptotic and necrotic cells (regions R3 and R4) is indicated. Results shown are representative of at least three independent experiments.

Chemotherapy plays a critical role in cancer management. Our previous studies demonstrated that VentX expression can be induced in malignant cells of hematopoietic origin by chemotherapeutic agents. To explore a potential role of VentX in chemotherapy of solid tumors, we asked whether VentX expression could be induced in cancer cells derived from solid tumors. HCT116 and H1299 cells were treated with 5-FU or DOX. Using qRT-PCR and western blot analysis, we found that VentX expression in HCT116 cells was significantly increased by 5-FU and DOX treatment. In comparison, VentX expression in H1299 cells can be induced only by DOX treatment (Figure 6a and 6b). In parallel to VentX induction, we showed that 5-FU and DOX treatment led to apoptosis in HCT116 cells, whereas DOX treatment led to apoptosis in H1299 cells (Figure 6c and 6d).

## DISCUSSION

Dysregulation of the cellular machinery that controls cell proliferation and differentiation undermines the pathogenesis of cancers. Current chemo- and radiation therapy focus primarily on disrupting proliferation of cancer cells and are often limited by significant side effects. Though targeting differentiation offers potential novel opportunities in cancer treatment [27], this approach is currently limited by our incomplete understanding of the cellular programs involved in the process.

In our recent investigation, we employed *Xenopus* embryogenesis as a model to dissect the mechanisms underlying concerted cell proliferation and differentiation during early embryogenesis. This allowed us 1) to identify the Xom/LEF/TCF pathway as a novel cell fate–determination pathway during vertebrate embryogenesis, and 2) to identify VentX, a human Xom homologue, as a novel Wnt antagonist (Figure 1a). VentX is expressed primarily in hematopoietic cells and exerts strong anti-proliferation and pro-differentiation effects through antagonizing canonical Wnt signaling, activating senescence pathways and inhibiting oncogenes such as Cyclin D1 [13] [15]. VentX gene is located on chromosome 10q23, a region frequently deleted in both solid and hematopoietic malignancies [16]. Our current studies show that VentX exerts strong inhibitory effects on the growth of cancer cells derived from solid tumors, similar to its effects on hematopoietic malignancies. In contrast, VentX exerts minimal inhibitory effects on the growth of transformed 293T cells (Figure 2). The findings are consistent with the prior notion of cancer's addiction to oncogenic signaling.

Our current studies also show that VentX induces apoptosis of cancer cells in a p53-independent manner (Figure 3 and 5). We found that VentX expression activates caspase 3 in cancer cells regardless of its p53 status (Figure 4). Our current conclusions are consistent with our prior findings that VentX activates both p53-dependent and p53-indepent senescence pathways [15]. Previous studies showed that p53 is mutated in more than 50% of cancers and that p53 mutations have been implicated in poor responses to cancer therapy and poor prognoses [24] [28]. As such, the discovery of VentX as a novel tumor suppressor may offer novel opportunities in treatment of cancers that carry p53 mutations.

Tissue-expression profiling showed that VentX is expressed primarily in hematopoietic cells; VentX expression in other tissues is low. However, we found that, similar to hematological malignancies, VentX expression in cancer cells derived from solid tumors can be induced by chemotherapeutic agents (Figure 6). It appears that types of cancer cells vary in their sensitivity to chemo-induction of VentX expression (Figure 6a and 6b). It is noteworthy that the VentX induction pattern in solid tumors correlates with clinical application of chemotherapeutic agents: 5-FU and DOX for colon cancers and DOX for lung cancers [29] [30]. A potential role of VentX in mediating chemotherapeutic effects was indicated by the finding that inhibition of VentX expression attenuated the cytotoxic effects of chemotherapeutic agents [15]. Whether VentX expression can be used to select effective chemotherapeutic regimens and to monitor cancer treatment remains to be explored further.

## MATERIALS AND METHODS

### Cell culture, transfection, chemotherapy

The human colorectal cancer lines HCT116 and SW480 and the human lung cancer cell lines H460 and H1299 came from America Type Culture Collection (Manassas, VA). The HCT116 (*p53*^−/−^) cell line was a gift of Dr. Bert Vogelstein, Johns Hopkins University. All cell lines were maintained at 37°C and 5% CO_2_. Cell culture media included McCoy's 5A (Invitrogen, Carlsbad, CA) for HCT116, HCT116 (*p53*^−/−^), and SW480 and RPMI-1640 (Invitrogen) for H460 and H1299. The cell culture media were supplemented with 10% fetal bovine serum (HyClone, Logan, UT), 100 units/ml penicillin, 100 μg/ml streptomycin, and 250 ng/ml amphotericin B (Mediatech, Hemdon, VA). Transfection was performed with Lipofectamine 2000 (Invitrogen) following the instructions of the manufacturer. The anticancer drugs used in the study, i.e., 5-fluorouracil (5-FU, 50 μg/ml) and doxorubicin hydrochloride (DOX, 0.4 μg/ml), were purchased from Sigma (ST. Louis, MO). All drugs were dissolved in DMSO and diluted to appropriate concentrations with cell culture media.

### Plasmids and constructions

GFP-VentX and GFP were subcloned and inserted into the pCS2+ vector by a polymerase chain reaction–based technique as detailed elsewhere [13].

### Confocal microscopy

HCT116 cells were seeded on glass chamber slides and transfected with GFP-VentX expression constructs. Twenty-four hours later, the cells were fixed with paraformaldehyde in PBS and counterstained with propidium iodide (PI, Sigma). After four washes in PBS for 5 min each, the slides were mounted and analyzed by confocal microscopy.

### Apoptosis and growth assay

Cells (including both attached and floating cells in the medium) were harvested and fixed in a solution containing a final concentration of 3.7% formaldehyde, 0.5% Nonidet P-40, and 10 μg/ml 4′,6-diamidino-2-phenylindole in PBS. Apoptosis was assessed through microscopic visualization of condensed chromatin and micronucleation. At least three independent experiments were carried out for each condition, and a minimum of 400 cells were counted in each measurement.

Cell growth was measured by 3-(4,5-dimethyl- thiazol-2yl)-5-(3-carboxymethoxyphenyl)-2-(4-sulfophenyl)-2*H*-tetrazolium (MTT) assay in 96-well plates (8,000 cells per well) using the CellTiter 96 AQueous One Solution (Promega, Madison, WI) following the instructions of the manufacturer. *A*_490 nm_ was measured using a VERSAmax Tunable Microplate Reader (Sunnyvale, CA). Vehicle-treated cells were used as 1 (100%). Each experiment was done in triplicate and repeated at least twice.

Cell apoptosis was measured using the Annexin V-FITC Apoptosis Detection Kit (eBioscience) following the manufacturer's instructions. In brief, one million HCT116 or H1299 cells were incubated with 40 μg/mL of 5-FU (Sigma), 0.4 μg/mL of DOX (Sigma), or DMSO control in 6-well plates at 37°C for the indicated times. Cells were washed with 1x PBS and then stained with Annexin V-FITC and 7-AAD (eBioscience). FACS analyses were performed on a FACScan (BD Biosciences), and data were analyzed using FlowJo software (Tree Star, Inc).

### Colony formation assay

Cells were transfected with Lipofectamine 2000 (Invitrogen) for 24 h in 100mm cell-culture dishes according to the instructions of the manufacturer. Then the cells were collected, and GFP-Vector- or GFP-VentX-positive cells were sorted using a FAC G4 sort flow cytometer (BD Biosciences). GFP-positive cells were plated in six-well plates at dilutions of 1 × 10^5^ cells per well. Cells were allowed to grow for 5-10 days before staining with Crystal Violet (Sigma). All experiments were repeated at least twice, and similar results were obtained in each trial.

### Xenograft tumors and tissue staining

All animal experiments were approved by the Institutional Animal Care and Use Committee at Harvard Medical School. Cells were transfected with Lipofectamine 2000 (Invitrogen) for 24 h in 100mm cell-culture dishes. Then the cells were collected, and vector-GFP- or GFP-VentX-positive cells were sorted using a FAC G4 sort flow cytometer (BD Biosciences). Xenograft tumors were established by s.c. injection of 1 × 10^5^ vector-GFP or GFP-VentX positive HCT116 (*p53*^+/+^) or HCT116 (*p53*^−/−^) cells into both flanks of 5- to 6-week-old female athymic nude mice (Simonsen Laboratories, Gilroy, CA). At the same time, 1 × 10^6^ unsorted cells, including GFP-positive and −negative cells, were also s.c. injected into other nude mice to establish an unsorted xenograft tumor model. Tumor growth was monitored thrice a week by caliper, and tumor volumes were calculated according to the formula (length × width^2^) / 2. Xenograft tumor tissue was immediately fixed in 10% neutral buffered formalin, then embedded in paraffin and sectioned. The sections were stained with hematoxylin and eosin (H&E) and then subjected to histological analysis. Terminal deoxyribonucleotidyl transferase–mediated dUTP nick-end labeling (TUNEL) staining was performed using recombinant terminal transferase (Roche, Indianapolis, IN) and dUTP-Alexa 594 (Molecular Probes) according to the instructions of the manufacturers and counterstained using 4′,6-diamidino-2-phenylindole. All images were acquired with a Nikon TS800 fluorescence microscope using SPOT camera imaging software.

### Cellular fractionation

Floating and attached cells were harvestedfrom two 60-cm^2^ dishes by centrifugation, resuspended in homogenization buffer (0.25 mol/L sucrose, 10 mmol/L HEPES pH 7.4, and 1 mmol/L EGTA), and subjected to 40 strokes of homogenization on ice in a 2-mL Dounce homogenizer. The homogenates were centrifuged at 1,000 × *g* at 4°C for 10 minutes to pellet nuclei. The supernatant was subsequently centrifuged at 14,000 × *g* at 4°C for 30 minutes to obtain cytosolic (supernatant) fractions.

### Real-time PCR

Total RNA was isolated using TRIzol reagent, and an equal amount of RNA was used for first-strand cDNA synthesis with SuperScript III First-Strand Synthesis System (Invitrogen) according to the manufacturer's protocol. We measured VentX cDNA with SYBR Green on a LightCycler (480 Real-Time PCR System; Roche). The relative mRNA levels were calculated using the comparative Ct method, using the housekeeping gene GAPDH as a normalizer. The primers used for the determination of mRNA levels of VentX and control GAPDH have been described previously [19].

### Western blotting

Western blot analysis was performed as described previously [19]. Briefly, total cell lysates as well as mitochondrial and cytosolic fractions were purified and separated by 4-20% Tris-Glycine Gel (Invitrogen, Carlsbad, CA) electrophoresis. For active caspase-3, PARP, and VentX analysis, total cells were extracted and separated by 4-20% Tris-Glycine Gel (Invitrogen) electrophoresis. Antibodies used included GFP (Santa Cruz Biotech, Santa Cruz, CA), Histone-1 (Ab-1, NeoMarkers, Fremont, CA), β-actin (Sigma), active caspase-3 (BD Biosciences), and PARP (Cell Signaling Technology, Boston, MA). Appropriate horseradish peroxidase-conjugated secondary antibodies were used to detect bound primary antibody antigen complex and developed with Western Lightning® Western Blot Chemiluminescence Reagent Plus (PerkinElmer, Boston, MA).

### Statistical analysis

Results are expressed as mean ± SD. Statistical analysis was carried out by ANOVA analysis in which multiple comparisons were performed using the method of least significant difference. Differences were considered significant if the probability of the difference occurring by chance was <5 in 100 (*P*<0.05).
